# Molecular insight into thymoquinone mechanism of action against *Mycobacterium tuberculosis*

**DOI:** 10.3389/fmicb.2024.1353875

**Published:** 2024-02-13

**Authors:** Grzegorz Jankowski, Rafał Sawicki, Wiesław Truszkiewicz, Natalia Wolan, Marcin Ziomek, Benita Hryć, Elwira Sieniawska

**Affiliations:** ^1^Chair and Department of Biochemistry and Biotechnology, Medical University of Lublin, Lublin, Poland; ^2^Student Research Group, Department of Pharmacognosy with Medicinal Plants Garden, Medical University of Lublin, Lublin, Poland; ^3^Department of Natural Products Chemistry, Medical University of Lublin, Lublin, Poland

**Keywords:** natural products, metabolomics, LC–MS, tuberculosis, lipids, biotransformation

## Abstract

Natural products are promising antimicrobials, usually having multiple and different cellular targets than synthetic antibiotics. Their influence on bacteria at various metabolic and functional levels contributes to higher efficacy even against drug-resistant strains. One such compound is a naturally occurring p-benzoquinone – thymoquinone. It is effective against different bacteria, including multidrug-resistant and extremely drug-resistant *Mycobacterium tuberculosis*. Its antibacterial mechanism of action was studied in several bacterial species except mycobacteria. To get an insight into the antimycobacterial activity of thymoquinone at the molecular level, we performed metabolomic and transcriptomic analyzes of bacteria exposed to this compound. The expression of genes coding stress-responsive sigma factors revealed that thymoquinone rapidly induces the production of *sigE* transcripts. At the same time, prolonged influence results in the overexpression of all sigma factor genes and significantly upregulates *sigF*. The metabolomic analysis confirmed that the antimycobacterial activity of thymoquinone was related to the depletion of NAD and ATP pools and the downregulation of plasma membrane lipids. This state was observed after 24 h and was persistent the next day, suggesting that bacteria could not activate catabolic mechanisms and produce energy. Additionally, the presence of a thymoquinone nitrogen derivative in the bacterial broth and the culture was reported.

## 1 Introduction

Natural products are promising antimicrobials that can influence bacterial cells on different metabolic and functional levels (Sieniawska and Georgiev, 2022). They usually have multiple cellular targets other than synthetic antibiotics. Therefore, they can inhibit the growth of susceptible and resistant bacterial strains (Alvarez-Martinez et al., 2020). Thymoquinone (TQ, 2-isopropyl-5-methyl-1,4-benzoquinone) is a naturally occurring p-benzoquinone with potent antimicrobial activity against different pathogenic bacteria (Chaieb et al., 2011; Goel and Mishra, 2018; Al-Khalifa et al., 2021; Dera et al., 2021; Wang et al., 2021), including *Mycobacterium tuberculosis* (Dey, 2015). TQ was proven active against multidrug-resistant and extremely drug-resistant mycobacterial strains (Dey et al., 2014), the emergence and spreading of which is a global problem in the treatment of tuberculosis (WHO, 2022). The discovery that TQ is highly effective in killing intracellular *M. tuberculosis* in mouse macrophages (Mahmud et al., 2017) suggested that this compound may be a potential drug candidate. Interestingly, the inhibition of bacterial growth was accompanied by diminished *M. tuberculosis-*induced secretion of pro-inflammatory cytokines from mouse immune cells (Mahmud et al., 2017). Already known strong anti-inflammatory activity of TQ (Woo et al., 2012) can be beneficial in restoring immunological balance disturbed by *M. tuberculosis*.

TQ’s antibacterial mechanism of action was studied in several bacterial species other than mycobacteria. It included the inhibition of cell adhesion to the glass slide surface (Chaieb et al., 2011), production of reactive oxygen species (Goel and Mishra, 2018), changes in the cell morphology, damage to the cytoplasmic membrane (Fan et al., 2021), membrane depolarization, and decrease in intracellular ATP concentration (Wang et al., 2021). However, there are no reports about the TQ mode of action in *M. tuberculosis*. Because TQ has promising activity, determining its molecular mechanism of action in *M. tuberculosis* is necessary.

The influence of TQ on bacterial cells can be monitored by metabolomics, which detects a large number of analytes and reflects the complexity of metabolic alterations. The significantly changed metabolites indicate targeted metabolic pathways and thus reflect the direct or indirect influence of the test compound on bacteria (Sieniawska and Georgiev, 2022). To get insight into the regulation of bacteria’s response to stress factors, a more comprehensive gene-metabolite approach is required (Sieniawska and Georgiev, 2022). Therefore, in this study, we applied both untargeted metabolomics and sigma factor gene expression analysis to determine the TQ mechanism of action in mycobacterial cells. Additionally, the stability and potential biotransformation of TQ were evaluated.

## 2 Materials and methods

### 2.1 Cells and substances

*Mycobacterium tuberculosis* H37Ra was purchased from the American Type Culture Collection (ATCC25177). It was grown on Löwenstein–Jensen slopes (BioMaxima, Lublin, Poland) for 2 weeks, then transferred to fresh Middlebrook 7H9 broth supplemented with 10% ADC and 0.2% glycerol (MilliporeSigma, St. Louis, United States). Thymoquinone of ≥98% purity was purchased from BIOKOM SA. (Janki, Poland). The purity was confirmed by HPLC analysis. Reference antibiotics, such as isoniazid, ethambutol, rifampicin, streptomycin, and ciprofloxacin were purchased from MilliporeSigma (St. Louis, United States), whilst resazurin (alamarBlue) solution was purchased from Invitrogen (Carlsbad, United States).

### 2.2 Bacteria exposure to thymoquinone

#### 2.2.1 Minimal inhibitory concertation determination

Before the metabolomic experiment, the minimal inhibitory concentration (MIC) of TQ was determined with the two-fold dilution method in the alamarBlue microplate assay adapted from Amin et al. (2009) and described in Sieniawska et al. (2021). Briefly, the stock solution of TQ was prepared in dimethylsulfoxide (DMSO), and then serial two-fold dilutions in the range of 256 to 2 μg/mL were obtained with the 7H9-S medium. The final DMSO concentration did not exceed 2% (v/v). Isoniazid, ethambutol, rifampicin, streptomycin, and ciprofloxacin were used as reference standards (in the concentration range from 16 to 0.001 μg/mL). The round bottom microwell plates were filled with 50 μL of inoculum adjusted to 0.5 McFarland standard [prepared as described in Sieniawska et al. (2021)] and 50 μL of TQ dilutions. Inoculated broth without TQ was a growth control, whereas broth without bacteria was a sterility control; 2% DMSO was included as well. Filled plates were sealed with an adhesive foil to prevent liquid evaporation, and the incubation lasted 8 days at 37°C. Then, 10 μL of resazurin solution was added to all wells, and incubation was continued for 48 h at 37°C. The MIC was defined as the lowest TQ concentration preventing visually evaluated blue to pink change.

#### 2.2.2 Effective sub-inhibitory dose determination

For the metabolomic experiment, the TQ dose has to be optimized because the culture density is much higher (10^9^ CFU/mL) in this experiment in comparison to the inoculum used for the MIC determination standardized procedure (1.5 10^6^ CFU/mL). Therefore, bacteria were grown in 300 mL cultures of Middlebrook 7H9 liquid medium supplemented with ADC enrichment for 4 to 5 weeks to obtain a density of 10^9^ CFU/mL. Then, serial two-fold dilutions of TQ, at the final concentration in the well, starting from 0.004 mg/mL to 1.024 mg/mL, were placed in a 96-well plate (50 μL) and supplemented with 50 μL of high-density culture and 15 μL of alamarBlue dye. The plate was sealed and incubated overnight at 37°C. The change in color of the alamarBlue from blue to pink was assessed. The effective dose value was confirmed by the lack of color change and defined as the effective sub-inhibitory dose.

#### 2.2.3 Bacteria exposure to thymoquinone

Four high-density bacterial cultures (109 CFU/mL) with a volume of 300 ml each were used in the experiment. Two (experimetal) cultures were supplemented with thymoquinone DMSO solution at the previously determined effective sub-inhibitory dose of 256 μg/mL. DMSO was added to the remaining two (control) cultures to a final concentration of 2%. All flasks were incubated in the same conditions (37°C with aeration, 100 rpm). The bacterial metabolism was stopped at two time points in both experimental and control cultures: after 24 h and after 48 h by adding cold methanol (−60°C, 1:1 v/v). Bacteria were centrifuged (30 min at 8000 rpm at 4°C), separated from the culture medium, washed twice with phosphate-buffered saline, collected, frozen in liquid nitrogen, and lyophilized. Dry samples were stored at −20°C. The metabolomic workflow was initially adopted from Halouska et al. (2013), and followed our previously published studies (Sieniawska et al., 2021).

### 2.3 *Mycobacterium tuberculosis* sigma factors expression analysis

For the sigma factor expression analysis, 2 mL of samples were taken after 3 and 48 h from high-density cultures exposed to TQ. The total RNA was isolated according to the manufacturer’s directions with the FastRNA Pro Blue Kit (MP Biomedicals, Santa Ana, United States, MP Biomaterials). The qPCR reactions were performed as described before (Sawicki et al., 2022). For the relative quantification of transcripts, targets were normalized to 16S rRNA. Primers used in the analyzes (Millipore, Sigma-Aldrich) are listed in Table 1. Relative mRNA quantification was calculated according to the delta–delta mathematical model.

**Table 1:** Primers used for sigma factor expression analysis.

Gene	Primer pair (5′–3′)
*sigA:*	FOR: GACGAAGACCACGAAGAC REV: TCATCCCAGACGAAATCAC
*sigB:*	FOR: CTCGTGCGCGTCTATCTGAA REV:AGCAGATGCTCGGCATACAA
*sigE:*	FOR: AACCCCGAGCAGATCTACCA REV: CTCGATGTCACACAGCACCA
*sigG:*	FOR: CGTCAATGAGCCTACGCAGA REV:GCGAAATTCCGTTCAGTCCG
*sig H:*	FOR: GCCGCTGTTTCTTGCGATAG REV: CCAGGAGACGATGGTGAAGG
*sig M:*	FOR: CGTCAGCAGTTGGTTGCAC REV: ACATCTTCTAGAGGGGCGGT
*sig L:*	FOR: CGTGATCCAGCGGTCCTAC REV: CAATCGCGACTTCACCGTTC
*sig J:*	FOR: GACCAGCCCGAGTATGAACC REV: ATCCCGACGTGACGTTTACC
*sig F:*	FOR: CCGCAGATGCAGTTCCTTGA REV: GGTCGGACTTCGTCTCCTTC
*sig D:*	FOR: AACAATCTCGTCCTTCAGCCG REV: CGAGATCCTCATTCTGCGTGT
*sig K:*	FOR: CCGCCACACCTCAAGATAGA REV: TCTACGACCACACCAAGTCG
*sig C:*	FOR: TTACCGCACTCGCCTTGTC REV: GGACAGATAGGCGACGAACC
*sig I:*	FOR: AAGACATGGTGCAAGAGGCA REV: ACGCCGACTTGATGTGATCC
*16S RNA*	FOR: ACTTCGGGATAAGCCTGGGA REV: AGCGCTTTCCACCACAAGAC

### 2.4 Metabolite extraction and analysis

#### 2.4.1 Biomass extraction

For the extraction of cellular metabolites, lyophilized bacterial biomass was weighted (ca 30 mg per sample, 6–10 replicates), poured with a mixture of methanol and water (1:1; v/v; 1.5 mL), and sonicated for 20 min at 35 kHz at ambient temperature (SONOREX RK, Bandelin, Berlin, Germany). After centrifugation (10 min at 13000 rpm, 4°C), the supernatant was collected, whilst the bacterial residue was extracted again. The combined supernatants were evaporated to dryness under reduced pressure at 30°C, and obtained residues were stored at -20°C before analysis (Sieniawska et al., 2021).

#### 2.4.2 Liquid chromatography-mass spectrometry analysis

For liquid chromatography-mass spectrometry (LC–MS) analysis, dry residues were dissolved in methanol–water (1:1; v/v; 1 mL) and filtered through 0.22-μm PTFE syringe filters. Analysis was performed on an Agilent 1,200 Infinity HPLC coupled with an Agilent 6530B QTOF spectrometer equipped with a Dual Agilent Jet Stream spray source (ESI) (Agilent Technologies, Santa Clara, CA, United States). A measure of 10 μL of each sample was injected into a Gemini® chromatographic column (3 μm i.d. C18 with TMS end-capping, 110 Å, 150 × 2 mm) supported by a guard column (Phenomenex Inc., Torrance, CA, United States). Chromatographic and mass spectrometry conditions followed the method previously described (Sieniawska et al., 2020). The solvents, water containing 0.1% formic acid (v/v) (A) and 0.1% formic acid in acetonitrile (v/v) (B) were pumped at 0.3 mL/min in the gradient: 5 min, 0% B; 20 min, 66% B; 35 min, 95% B. The MS detection was performed in positive ionization mode, with drying gas temperature: 350°C, drying gas flow: 12 L/min, sheath gas temperature: 400°C, sheath gas flow: 12 L/min; nebulizer pressure: 40 psig, capillary V (+): 4000 V, and skimmer 65 V. MS and MS/MS acquisition rates were 2 spectra/s in a scan range of 50–1700 m/z, recorded in the collision energy range of 10 and 30 eV. For accurate online mass calibration, the standard masses (121.0508 and 922.0097) were injected directly into the ion source.

#### 2.4.3 Cell wall hydrolysis and thin layer chromatography analysis of mycolic acids

For the extraction of mycolic acids bound to arabinogalactan, 50 mg of lyophilized test and control bacterial biomass were prepared. The procedure of hydrolysis and methylation of mycolic acids was adopted from Sambandan et al. (2013) and performed with some modifications. Biomass was placed in a glass tube and suspended in 2 mL of water. Then, 2 mL of 40% tetrabutylammonium hydroxide was added, and the suspension was heated at 100°C for 20 h. After cooling, 200 μL of iodomethane and 4 mL of dichloromethane were added, and the mixture was shaken for 1 h at room temperature. The organic phase was collected, washed with 2 mL of 1 M HCL and then with 2 mL of water, and evaporated. The obtained residue was dissolved in 400 μL of dichloromethane. The extracts were applied onto a silica gel plate (Si60 F254, Merck) in the same volumes. The plate was developed three times with a mixture of hexane and ethyl acetate (95:5 v/v). After drying, it was sprayed with a 10% solution of phosphomolybdic acid and heated at 100°C until spots appeared.

### 2.5 Data processing

For metabolite analysis, raw data obtained from LC–MS acquisition were converted in Mass Hunter Qualitative Analysis software (version B.07.00; Agilent Technologies, Santa Clara, CA, United States) to mzDATA format. Open-source XCMS online software (version 3.7.1) for feature extraction was used. CentWawe and Obiwarp methods for feature detection and retention time correction were applied. The intensity threshold was set at 500; features were retained only if present in at least six replicates, and in pairwise analysis, they were considered statistically significant at a value of p of <0.01 (Welch t-test). MS-LAMP software^1^ with an integrated *Mycobacterium tuberculosis* (M. tb) Lipidome database was used to annotate mycobacterial lipids as singly protonated ions (M + H) + with an allowed 0.05 m/z mass difference, whilst other compounds were tentatively identified in MetaboAnalyst5.0^2^ with the MS Peaks to Pathways module with a mass accuracy of 5 ppm. Only compounds confirmed by EIC chromatograms and MS/MS spectra were further submitted for enrichment analysis.

### 2.6 Thymoquinone stability

#### 2.6.1 Incubation conditions and detection of biotransformation products

For the evaluation of stability and potential biotransformation, TQ (256 μg/mL) was added to 10 mL of culture broth (CB) and to 10 mL of culture broth inoculated with bacteria (CBI). Both flasks were kept at 37°C with agitation. A measure of 1 mL of sample was taken from both flasks at time zero, after 24 h and after 48 h. The samples were filtered through 0.22 μm PTFE syringe filters and subjected to LC–MS analysis, which was performed using the same method as described in Section 2.4. Detection was performed in MS and in UV (*λ* = 254 nm).

#### 2.6.2 Quantification of thymoquinone

For quantification, TQ (350 μg/mL) was added to 10 mL of culture broth (CB), incubated, and sampled as described above. HPLC analysis was performed on the Shimadzu HPLC system equipped with an automatic degasser (DGU-20A 3R), a quaternary pump (LC-20 AD), an autosampler (SIL-20A HT), and a DAD detector (SPD-M20A), which were controlled and monitored by LabSolutions 5.51 software (Shimadzu, Japan). A measure of 10 μL of the sample was injected into the chromatographic column Eclipse XDB-C18 (250 mm × 4.6 mm, 5 μm) at 25°C. A measure of 1% aqueous acetic acid (A) and acetonitrile (B) was pumped at 1 mL/min in the gradient: 60% B for 7 min, 60–95% B for 2 min, 95–60% B for 5 min. *λ* = 254 nm was set for the detection. The external calibration curve was prepared in the range of 5–300 μg/mL of the TQ reference standard.

### 2.7 Statistical analysis

The pairwise comparison of the test and control groups in the metabolomic experiment was based on the Welch t-test. The results were considered statistically significant at a *p*-value of <0.01. Other comparisons were based on the mean ± standard deviation of the mean (SD). The observations of lipid levels and gene expression results were calculated as fold changes. In the metabolomic experiment, 6–10 replicates were run, whilst other experiments were performed in triplicate.

## 3 Results

### 3.1 Bacteria susceptibility and their metabolite profile after exposure to TQ

In the first step, we determined the susceptibility of mycobacteria to thymoquinone. The observed MIC value equaled 32 μg/mL. This value was obtained for a standardized amount of bacteria per mL (1.5 × 10^6^ CFU/mL); however, metabolomic experiments require high-density cultures (10^9^ CFU/mL), as indicated by Halouska et al. (2013). The stressing compound should be used in a dose large enough to affect the cellular metabolome relative to untreated cells but should not induce cell death (Halouska et al., 2013). The drug concentration between 1 and 24 times the MIC should be used to identify an optimal drug dosage (Halouska et al., 2013). Hence, we used the AlamarBlue assay to experimentally determine the effective sub-inhibitory dose of TQ. AlamarBlue is a tetrazolium compound reduced by metabolically active cells to a purple formazan product (Amin et al., 2009). The highest concentration of TQ, which did not cause the change from pink to blue in the high-density culture, was 256 μg/mL, and this concentration was used in the metabolomic experiment. In the metabolomic experiment, bacteria should be allowed to grow with the stressing agent for at least one generation before harvesting (Halouska et al., 2013). In this study, *M. tuberculosis* was incubated with TQ for 24 and 48 h. These correspond to approximately one and two replication cycles of slowly growing mycobacteria, which divide once per 18–24 h (Wanger et al., 2017).

**Figure 1:**
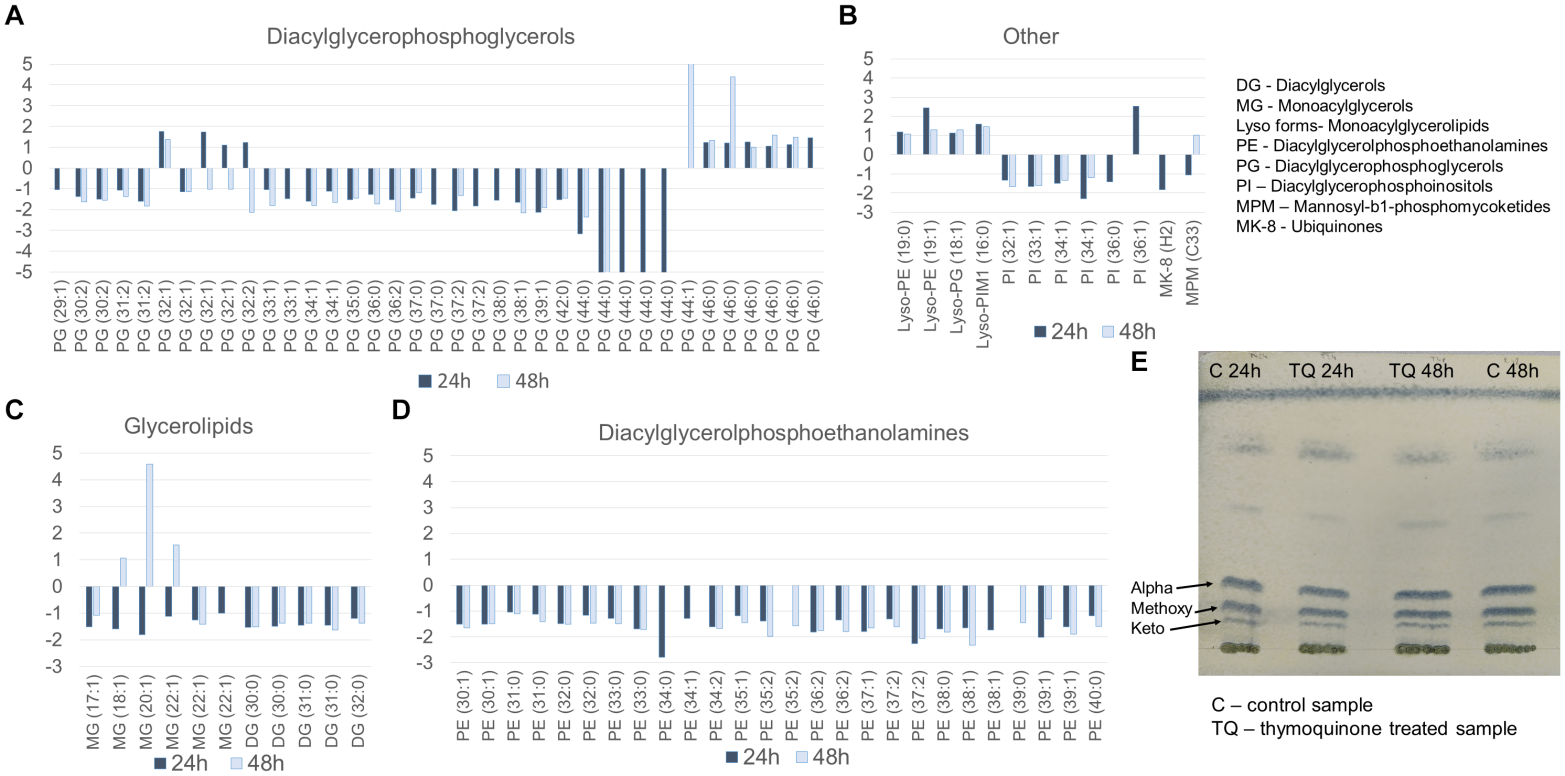
Lipid profile after bacteria exposure to thymoquinone for 24 and 48 h. **(A–D)** Fold change of lipids in classes (test group in relation to the control group); the pairwise comparison was based on a Welch t-test, and the results were considered statistically significa at a *p*-value of <0.01. **(E)** mycolic acid methyl esters separated into alpha, methoxy, and keto subclasses on a TLC Si60 plate, developed with a mixture of hexane–ethyl acetate (95.5 v/v) derivatized with a 10% solution of phosphomolybdic acid (100°C). Control group – untreated bacteria.

Untargeted LC–MS analysis was performed to identify dysregulated metabolites. The changes in the levels of lipids were presented as fold changes in relation to the control group and were considered statistically significant with a value of p of <0.01. The exposure of mycobacteria to TQ resulted in the downregulation of almost all detected lipid classes. The exceptions were individual glycerolipids, glycerophospholipids, and several lysoforms, which are monoacylated glycerophospholipids (Figures 1A–D). The downregulation was observed after the first and second day of the exposure, suggesting a slight (small fold change) but long-lasting influence of TQ. Additionally, the hydrolysis of the bacterial cell wall and the methylation of liberated mycolic acids, which were visualized on TLC plates, revealed that TQ does not influence the mycomembrane, as mycolic acid fractions in the test and control samples after both incubation times were similar (Figure 1E).

**Figure 2:**
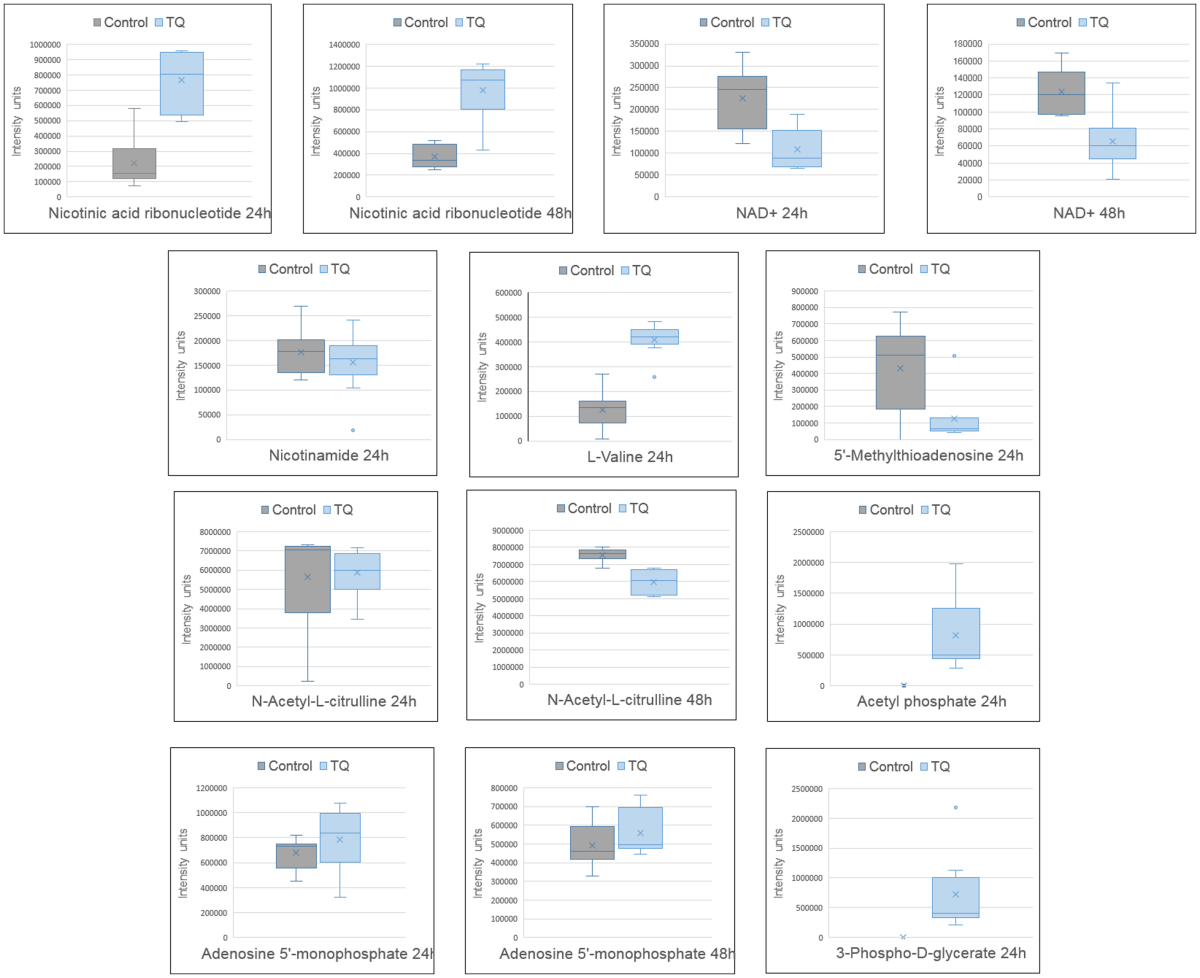
Metabolite profile after bacteria exposure to thymoquinone. Boxplots of significantly changed metabolites (*p* < 0.01) after 24 h and 48 h of exposure. The pairwise comparison was based on the Welch *t*-test. Control group – untreated bacteria.

**Figure 3:**
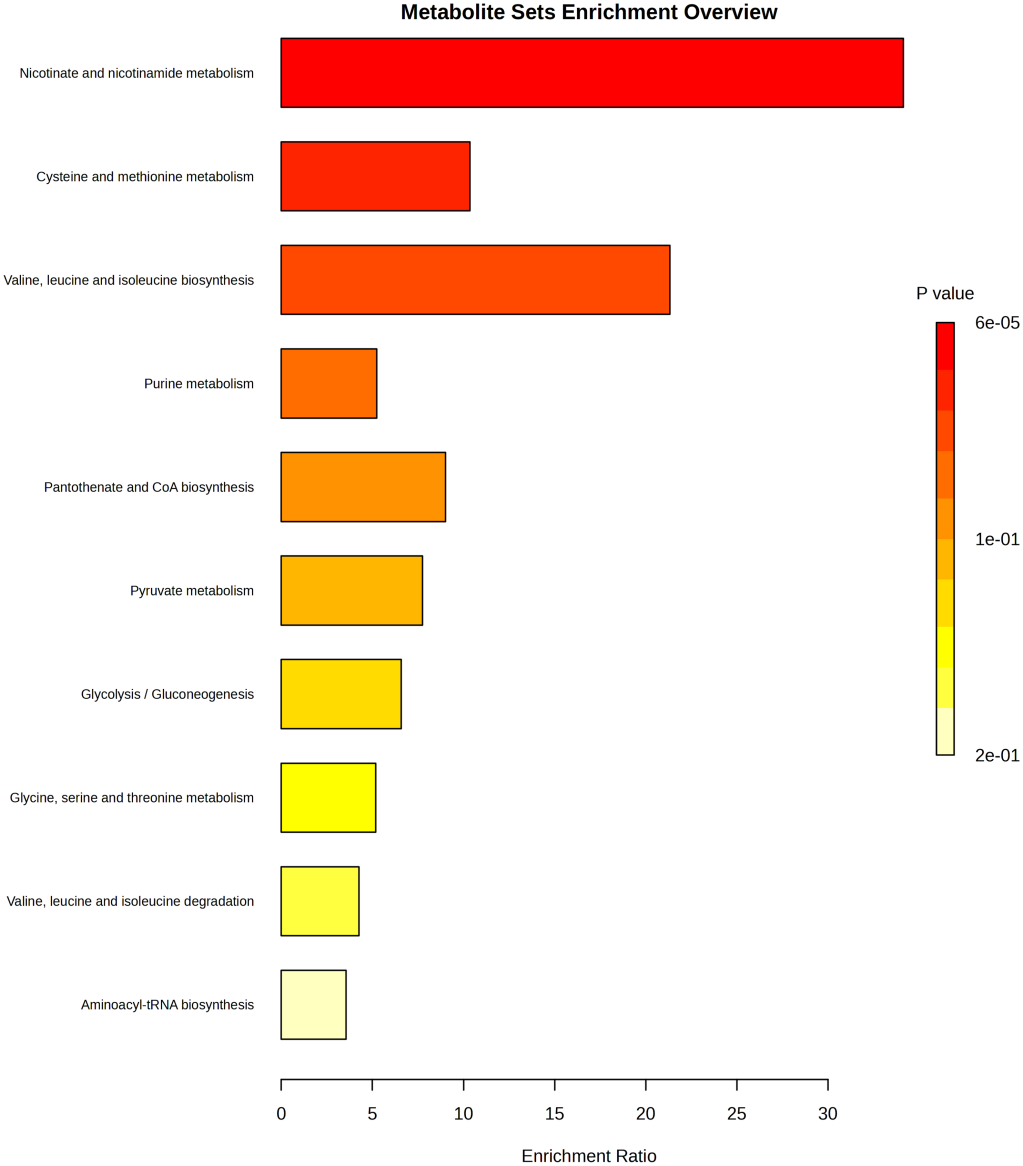
Enrichment analysis showing general metabolic pathways influenced by thymoquinone. Analysis was performed for the significantly changed metabolites presented in Figure 2. The color intensity corresponds to the value of p; enrichment ratio is computed by hits/expected, where hits = observed hits; expected = expected hits.

Untargeted LC–MS analysis enabled the description of other compounds that were influenced by bacteria after their exposure to TQ. Molecules upregulated in test samples were nicotinic acid ribonucleotide, L-valine, acetyl phosphate, 3-phospho-D-glycerate, and adenosine 5′-monophosphate, whereas for nicotinamide adenine dinucleotide, 5′-methylthioadenosine, nicotinamide, and N-acetyl-L-citrulline, downregulation was noticed (Figure 2). The changes in the intensities of these metabolites were presented as box plots and were considered statistically significant with a value of p of <0.01. Indicated compounds were subjected to enrichment analysis, which showed in what metabolic processes they are involved (Figure 3). Nicotinic acid and nicotinamide metabolism had the highest enrichment ratio (almost 35) and statistical significance. It was followed by cysteine and methionine metabolism, and valine, leucine, and isoleucine biosynthesis. A lower score but still high significance was obtained for purine metabolism (Figure 3).

### 3.2 Sigma factors genes expression after bacteria exposure to TQ

**Figure 4:**
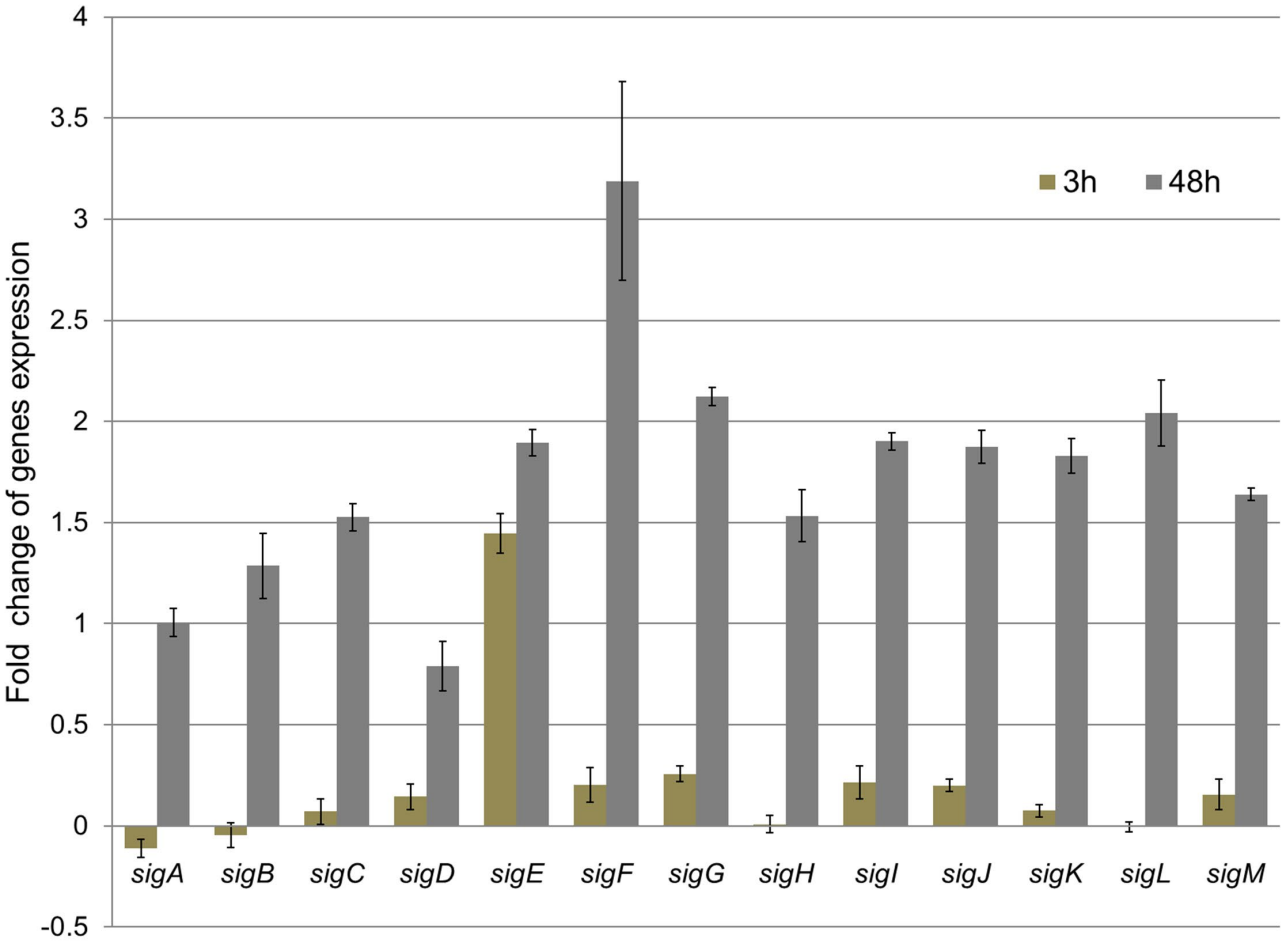
Relative fold change of *M. tuberculosis* sigma factors gene expression after 3 h and 48 h exposure to thymoquinone, normalized to 16SmRNA (control). The value of 1 obtained for the control was subtracted from all gene expression results.

To track mycobacteria response to TQ at the transcriptional regulatory level, we measured the shifts in transcripts of 13 genes encoding sigma transcription factors. This group of regulatory genes controls the remodeling of metabolism in the adaptive bacterial response to environmental conditions (Rodrigue et al., 2006). Therefore, the evaluation of expression levels of sigma factors provides a global view of the bacterial response to compounds inhibiting their growth. The bacteria harvest time for gene expression analysis was set at 3 and 48 h. The first time point gave an insight into the rapid bacterial response at the genetic level. These rapid transcriptomic changes preceded the metabolic rearrangement. The second analysis performed after 48 h of incubation with TQ proved that transcriptomic changes are dynamic in time and can be correlated with metabolomic dysregulations. Bacteria exposed to TQ responded in a time-dependent manner. After 3 h, a significant upregulation of *sigE* was observed. The fold change of that gene was up to five times higher than the fold change of other monitored genes. Later, after 48 h, all the *sig* genes were significantly overexpressed, with noticeable dominance of the *sigF* transcript (Figure 4).

### 3.3 TQ stability

**Figure 5:**
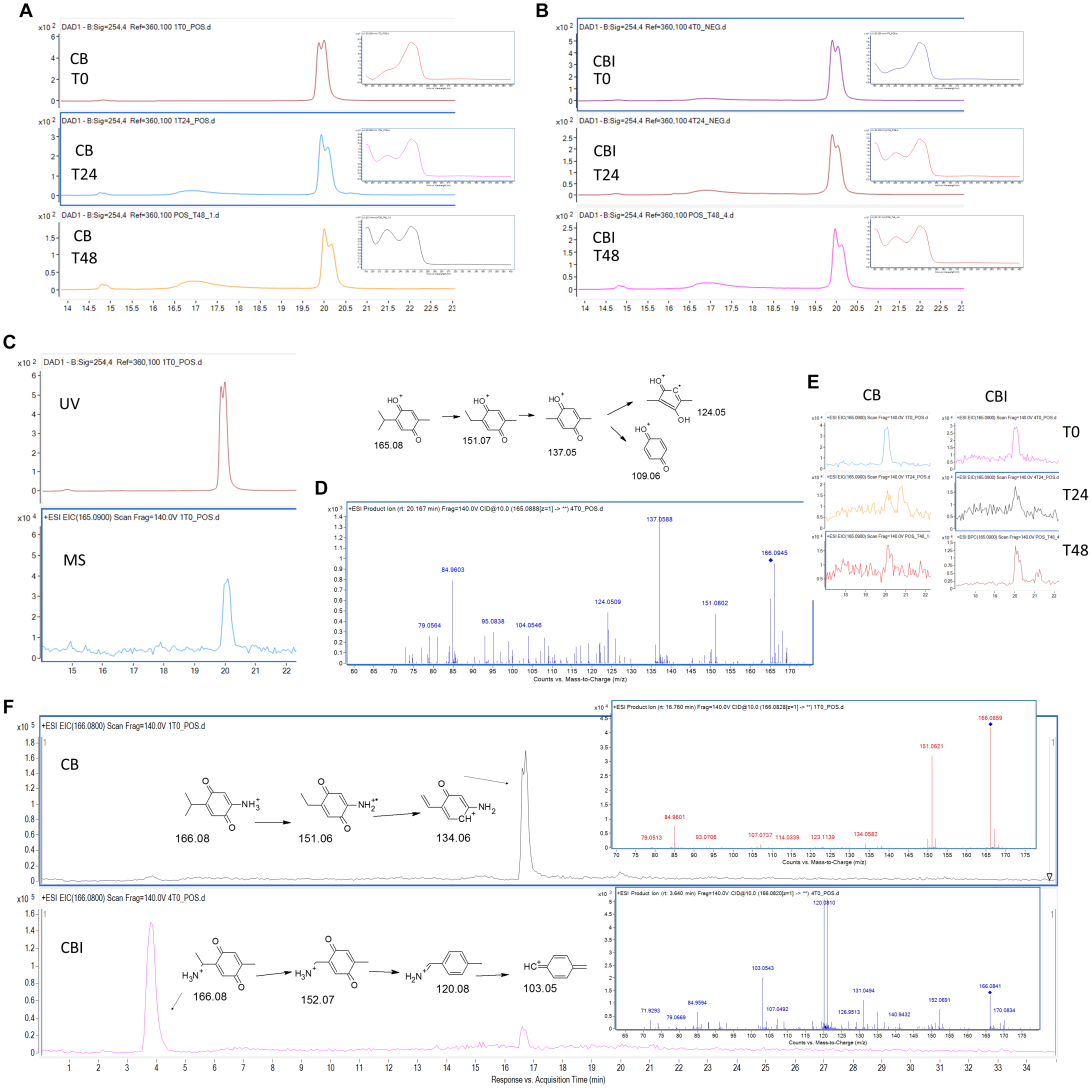
Thymoquinone (TQ) stability and biotransformation. DAD chromatograms (*λ* = 254 nm) and UV spectra of TQ in CB **(A)** and in CBI **(B)** over incubation time; TQ signal on DAD and EIC chromatograms, tR: 20.1 min **(C)**; MS/MS spectrum and proposed fragmentation pathway of TQ **(D)**; EIC chromatograms of TQ in CB and CBI over incubation time **(E)**; EIC chromatograms, MS/MS spectra, and proposed fragmentation pathways of TQ derivatives in CB and CBI **(F)**. CB, culture broth; CBI, culture broth inoculated with bacteria; T0, starting time point; T24, after 24 h; T48, after 48 h; tR, retention time.

The LC-DAD-MS analysis showed a similar profile of TQ stability in CB and CBI (Figures 5A–E). Regardless of the presence of bacteria, TQ content was decreasing over time, which was proven by lowered signal intensity on DAD and EIC chromatograms and by observed changes in UV spectra. HPLC analysis confirmed diminished TQ concentration and peak purity, especially after 48 h (Table 2). The pseudomolecular ion of TQ, m/z 165.08, was observed at 20.1 min (Figure 5D). Its usual degradation products, hydrothymoquinone, thymoquinone dimer, or thymol, were not confirmed. Interestingly, two pseudomolecular ions of m/z 166.08 were detected. In the CB sample, m/z 166.08 was present at 16.7 min, whilst in the CBI it was accompanied by a second ion at 3.6 min (Figure 5F). Despite the same m/z value and molecular formula (C9H11NO2), the observed ions were characterized by different MS/MS spectra and fragmentation patterns (Figure 5F). Isomer 2-amino-5-(propan-2-yl)cyclohexa-2,5-diene-1,4-dione was present in culture broth without bacteria, whilst 2-(1-aminoethyl)-5-methylcyclohexa-2,5-diene-1,4-dione appeared in the inoculated sample, suggesting that this isomer may be a product of cellular biotransformation.

**Table 2:** Thymoquinone stability in the culture broth.

Time point (h)	Concentration (μg/mL)	% of initial concentration	Peak purity (%)
0	351.14	100	94.5
24	343.57	97.84	89.4
48	224.15	63.83	84.7

## 4 Discussion

### 4.1 Changes in the sigma factors regulatory network

*M. tuberculosis* responds to different environmental stimuli through the transcription of specific gene sets, which create a three-level network hierarchy of 13 sigma factors grouped into 5 communities (Chauhan et al., 2016). The bacteria’s exposure to TQ first caused a rapid (after 3 h) and selective shift in the number of transcripts of a major stress-responsive sigma factor, the sigE gene (Jensen-Cain and Quinn, 2001; Raman et al., 2001). sigE is induced via surface stress (Flentie et al., 2016) and upregulates mprA and mprB genes responsible for sensing cell envelope integrity and transferring stress information to the bacterial cytoplasm (Bretl et al., 2014). Additionally, sigE participates in the response to xenobiotic stimuli, oxidative stress, and regulation of fatty acids and lipid metabolism (Karp et al., 2019). The early upregulation of *sigE* expression may suggest that TQ rapidly disturbs the integrity of the mycobacterial cell envelope. The later (after 48 h) overexpression of all sigma factor genes caused by TQ indicates the activation of different genetic pathways aiming to overcome the influence of the stressing agent. Under the influence of TQ bacteria increased the transcription of genes (*sigC, sigD, sigE, sigG, sigH, sigI, sigJ, sigK, sigL,* and *sigM*) coding sigma factors controlling cell envelope synthesis, secretory functions, and periplasmic protein repair and degradation increased (Lee et al., 2008). Interestingly, the expression of *sigF* after 48 h was much higher than that of other genes. In *M. tuberculosis*, this gene was associated with a stationary phase/stress response related to nutrient depletion (Demaio et al., 1996). The observed significant upregulation of *sigF* suggests that TQ caused the exhaustion of energy resources in bacterial cells.

### 4.2 Metabolic perturbations caused by TQ

The characteristic feature of mycobacteria is the very high content of lipids in their cell envelope. These abundant and diverse lipids comprise approximately 40% of the dry cell mass, and together with sugar conjugates, they form an impermeable barrier, strongly contributing to bacterial resistance (Crellin et al., 2013). To investigate the potential influence of TQ on the state of this barrier, we performed a metabolomic analysis of the bacterial lipid profile. The observed downregulation of phosphatidylethanolamines, phosphatidylinositols, and phosphatidylglycerols (Figure 1), which constitute the plasma membrane (Crellin et al., 2013), suggests somewhat diminished production of these molecules caused by TQ. Additionally, only a few “lyso” forms, being partial degradation products of phosphoglycerolipids (Sahonero-Canavesi et al., 2019), were upregulated, indicating that rather reduced metabolic activity than significant degradation of the cell membrane can be considered. Interestingly, the upregulation or downregulation of the mycolic acid fraction was not observed under TQ influence. These long-chain α-branched, β-hydroxylated fatty acids covalently linked to the arabinogalactan layer (Crellin et al., 2013) had a profile similar to the control (Figure 1), meaning that TQ does not target mycomembrane. The changes observed in plasma membrane lipids may be associated with their quite rapid turnover. For example, the diacylglycerophosphoinositol pool is renewed within 7 h within mycobacterial cells (Haites et al., 2005). Therefore, the fast cycle of degradation and renewal gives a better chance to observe even slight dysregulation due to the affected metabolic activity of the cell.

TQ caused significant upregulation of nicotinic acid ribonucleotide (nicotinic acid mononucleotide, NaMN) and significant downregulation of nicotinamide adenine dinucleotide (NAD). In *M. tuberculosis*, NaMN is synthesized from aspartate, and after the addition of the adenyl group from ATP, it forms nicotinic acid adenine dinucleotide (NaAD). NaAD can be further converted into nicotinic acid adenine dinucleotide phosphate (NAADP), a Ca^2+^-mobilizing second messenger synthesized in response to extracellular stimuli, or into NAD and nicotinamide adenine dinucleotide phosphate (NADP), ubiquitous co-enzymes. NaMN is a unique checkpoint intermediate shared by all *de novo*, salvage, and recycling pathways (Rodionova et al., 2015). The upregulation of this intermediate may suggest an increased need for NAADP or NADP production under the influence of TQ. Importantly, the level of nicotinamide in test samples was very slightly but statistically significantly downregulated, confirming rather increased production reactions than the degradation of NAD because nicotinamide is a degradation product of NAD (Kasarov and Moat, 1972; Rodionova et al., 2015). Moreover, lowered levels of NAD in test samples indicate the depletion of NAD in bacterial cells after exposure to TQ. In *M. tuberculosis*, the result of NAD depletion is a sharp suppression of metabolic flux in multiple NAD(P)(H)-dependent pathways, including the synthesis of many amino acids (serine, proline, and aromatic amino acids) and fatty acids, the central metabolism of carbon, and energy production (Rodionova et al., 2014). In this study, lowered NAD levels were correlated with the downregulation of cell membrane lipids, confirming diminished metabolic activity as a result of TQ influence. Because accumulated NaMN was not converted into NAD, it can be hypothesized that TQ may possibly target metabolic steps between NaMN and NAD.

TQ caused a very pronounced downregulation of 5′-methylthioadenosine (MTA), a by-product of polyamine synthesis (spermine and spermidine). The reaction consumes S-adenosylmethionine (originating from methionine) and releases MTA. Because MTA is a potent inhibitor of polyamine biosynthesis and transmethylation reactions, it has to be removed from the methionine and adenine salvage pathways. Thus, MTA is used to recycle sulphur (in methionine) and AMP from adenine (Avila et al., 2004; Karp et al., 2019). If there is a lot of MTA, the need to convert adenosine to AMP is less (Albers, 2009). The detected lowered levels of MTA after bacteria exposure to TQ may mean increased polyamine biosynthesis or MTA extensive degradation. The co-observed raised level of AMP suggests a second scenario. The increasing levels of AMP (and ADP) are naturally accompanied by decreasing levels of ATP (Carling et al., 2011). Furthermore, acetyl phosphate, which was detected to be upregulated in test samples, is a product of ATP degradation (Karp et al., 2019). These indicate that TQ causes energy depletion in mycobacterial cells. This state was observed after 24 h and was persistent the next day, meaning that bacteria were unable to activate catabolic mechanisms, which generate ATP. This may be a consequence of NAD depletion and vice versa because the conversion of NaMN into NaAD needs ATP.

Additionally, the observed downregulation of lipids in the test samples was consistent with L-valine accumulation. L-Valine is a branched-chain amino acid that is degraded to produce isobutyryl-CoA, which is involved in branched-chain fatty acid biosynthesis. L-Valine degradation also yields propionyl-CoA, which supplements polyketide biosynthesis (Karp et al., 2019; Borah et al., 2021). The NAD depletion-induced decreased metabolic activity, including diminished flux for lipid biosynthesis, may contribute to the L-valine accumulation under TQ influence.

In the scientific literature, the alterations in the ATP levels and the NAD/NADH and NADP/NADPH ratios were correlated with the disruption of the mycobacterial membrane upon the action of bactericidal peptide laterosporulin10 (Baindara et al., 2016). It was confirmed that latersporulin10 kills the Mtb H37Rv strain very efficiently, and the mechanism of action was related to the disruption of the cell membrane (Baindara et al., 2016). In this study, some correlation between targeted cellular membranes and disturbed energetic homeostasis can be observed. As indicated above, the early upregulation of *sigE* expression may suggest that TQ rapidly affects the integrity of the mycobacterial cell envelope. Furthermore, the downregulation of lipids in the cell membrane confirms the possibility that electron transfer reactions within the cytoplasmic membrane might be altered, resulting in energy depletion. This state was long-lasting, as confirmed by the high upregulation of *sigF* after 48 h, which is associated with a stationary phase/stress response related to nutrient depletion (Demaio et al., 1996). Therefore, our study is in agreement with the statement that changes in energy pools are correlated with the rearrangement/disruption of the bacterial membrane.

### 4.3 Thymoquinone stability

The results show that regardless of the presence of bacterial cells, the TQ content decreases over time in the culture broth (Table 2). After the second day, TQ concentration lowered significantly, meaning that TQ is not stable in incubation conditions (elevated temperature, aeration, and light exposure). The lowering of the TQ content was manifested by the changed sample color, which became violet-brown, suggesting the formation of some metabolite. Indeed, mass spectrometry analysis revealed the presence of intense ions with the m/z value being one unit higher than the TQ ion. The detected derivative is nitrogen-substituted TQ analog (C9H11NO2) (Figure 5) The observed m/z value of 166.08 indicates that the amine group replaced the methyl group. The addition of an amine group in place of oxygen would produce an ion with a m/z of 166.12; however, this was not recorded. The nitrogen substitution occurred very fast because the metabolite was observed at the initial incubation time. Previously, this TQ derivative was obtained by Johnson-Ajinwo et al. (2018) by 2 h incubation at room temperature in the presence of diethylamine/ethylamine, water, and methanol (Johnson-Ajinwo et al., 2018). In our study, TQ was added to bacterial broth containing monosodium glutamate, which can be a source of organic nitrogen (Dubos and Middlebrook, 1947). The sample inoculation with bacteria resulted in the formation of an isomeric TQ analog, which was observed at the other retention time (Figure 5). This suggests that bacteria are able to change the position of the amine group in the TQ structure, converting 2-amino-5-(propan-2-yl)cyclohexa-2,5-diene-1,4-dione into 2-(1-aminoethyl)-5-methylcyclohexa-2,5-diene-1,4-dione. Nevertheless, since after 24 h the concentration of TQ in the test sample was more than 97% of its initial value, we can assign the observed metabolic perturbations to TQ influence. However, we also cannot exclude any possible activity of its detected nitrogen derivatives.

## 5 Conclusion and limitations of the study

The metabolomic analysis performed revealed that TQ influenced mycobacterial cells at the molecular level. Its mechanism of action was related to NAD depletion, the consequence of which was a suppression of metabolic flux in multiple NAD(P)(H)-dependent pathways, including the observed diminished synthesis of lipids and energy production. Bacteria were unable to activate catabolic mechanisms, which generate ATP. The influence of TQ was long-lasting, as observed after 24 h and 48 h. Moreover, the expression of stress-responsive sigma factor genes showed that TQ rapidly (after 3 h) disturbs the integrity of the mycobacterial cell envelope and later (after 48 h) activates different genetic pathways, related mainly to cell envelope synthesis, secretory functions, and periplasmic protein repair and degradation. Notably, the observed significant upregulation of *sigF* suggests that TQ causes the exhaustion of energy resources in bacterial cells; thus, transcriptomic observations were consistent with metabolomic ones. The TQ mechanism of action described in this study is in agreement with previously reported damage to the cytoplasmic membrane (Fan et al., 2021), and a decrease in intracellular ATP concentration (Wang et al., 2021). It is worth noticing that the stability of TQ in bacterial broth was limited and, after 48 h, significantly lowered. Nevertheless, since after 24 h the concentration of TQ in the test sample was more than 97% of its initial value, we can assign the observed metabolic perturbations to TQ influence. The observed nitrogen derivative of TQ appeared regardless of the presence of bacteria in the broth; however, bacteria performed the isomerization of this product.

The untargeted metabolomic analysis gives the opportunity to globally monitor the metabolic changes upon bacterial exposure to TQ. This approach is not based on any preliminary assumptions about the mechanism of action but is focussed on the observations of bacteria’s response to the stressing agent. This gives a chance to monitor different metabolic perturbations in bacterial cells; however, there are also some limitations. Metabolomics analyzes only “small” metabolites; hence, any interactions at the protein level are not taken into consideration, even though they may exist. Moreover, performed observations are “static” because the metabolism is stopped at certain time points, so any possible fluctuations between these time points are missed.

## Data availability statement

The datasets presented in this study can be found in online repositories Zenodo: 10.5281/zenodo.10580402.

## Author contributions

GJ: Investigation, Writing – original draft. RS: Conceptualization, Funding acquisition, Investigation, Methodology, Visualization, Writing – original draft, Writing – review & editing. WT: Investigation, Writing – original draft. NW: Investigation, Writing – original draft. MZ: Investigation, Writing – original draft. BH: Investigation, Writing – original draft. ES: Conceptualization, Funding acquisition, Investigation, Methodology, Project administration, Supervision, Visualization, Writing – original draft, Writing – review & editing.

## Footnotes

^1^ http://ms-lamp.igib.res.in

^2^ https://www.metaboanalyst.ca/
